# Supplementary material for: Genetic determinants and phenotypic consequences of blood T-cell proportions in 207,000 diverse individuals
Source: Nat Commun. 2024 Aug 7;15:6732. doi: 10.1038/s41467-024-51095-1 (PMC11306580; doi:10.1038/s41467-024-51095-1)
Supplement: Supplementary file 1 — Supplementary Information [file 41467_2024_51095_MOESM1_ESM.pdf]

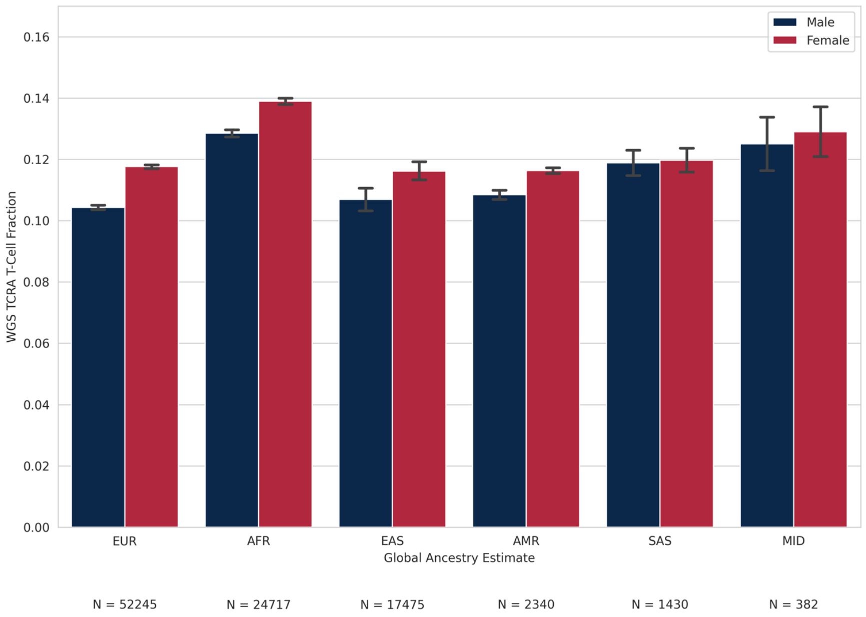


**Supplementary Figure 1: All of Us global ancestry distributions.** Mean All of Us WGS T-cell fractions, stratified into six super populations using global ancestry estimates (N=98,589). Error bars represent 95% CI.

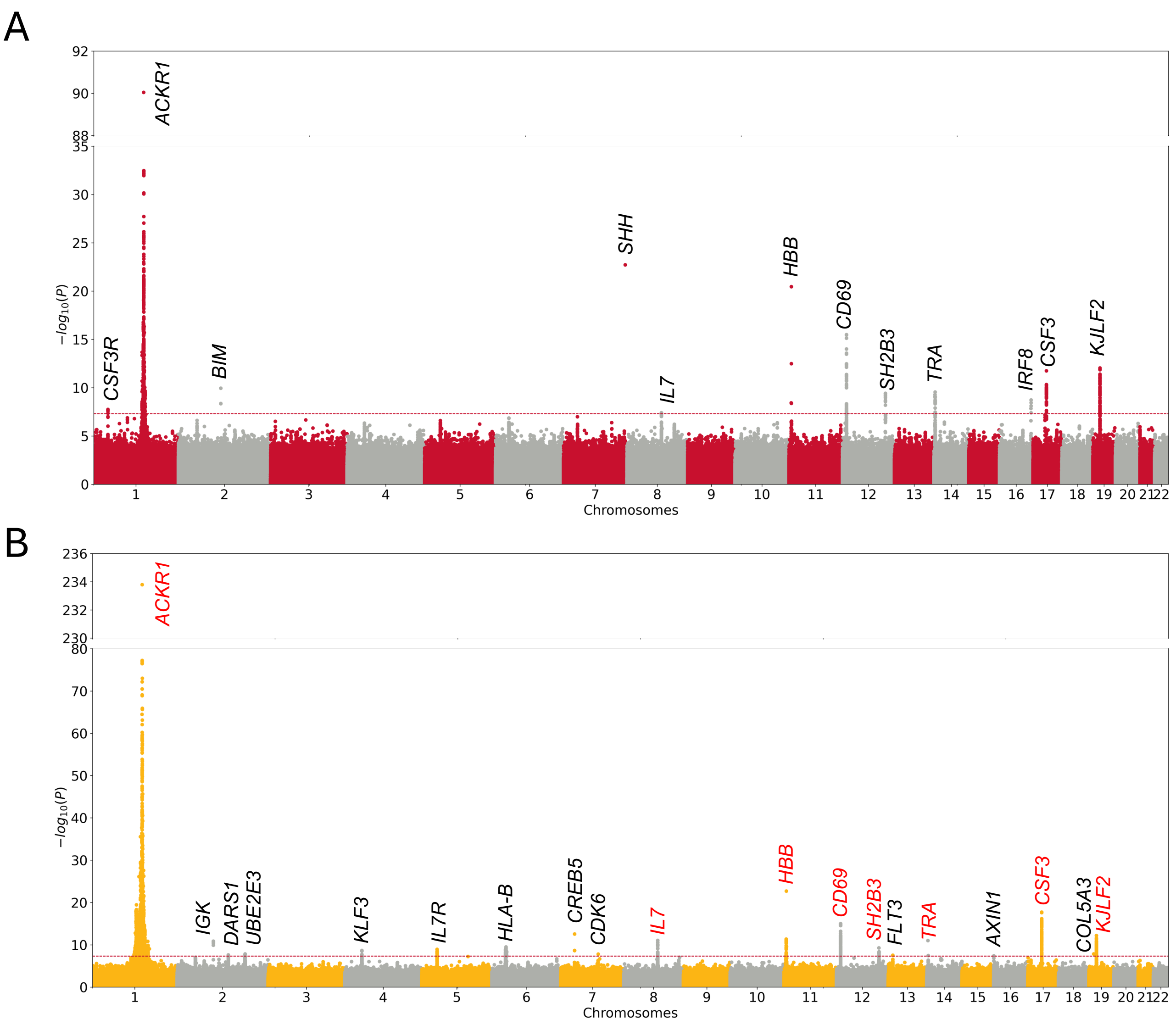


**Supplementary Figure 2: Individual cohort genome-wide association studies.** a) Single-variant association analyses of genetically estimated T-cell fraction identified 12 genome-wide significant loci. Two-side association testing performed using SAIGE (N=86,017). The dashed red line represents $5 \times 10^{-8}$, our Bonferroni multiple-hypothesis correction p-value threshold for significance. b) Replication single-variant association analyses of genetically estimated T-cell fraction identified 11 novel and 8 (in red) replicated genome-wide significant loci. Two-side association testing performed using REGENIEv3.2 (N=95,551). The dashed red line represents $5 \times 10^{-8}$, our Bonferroni multiple-hypothesis correction p-value threshold for significance.

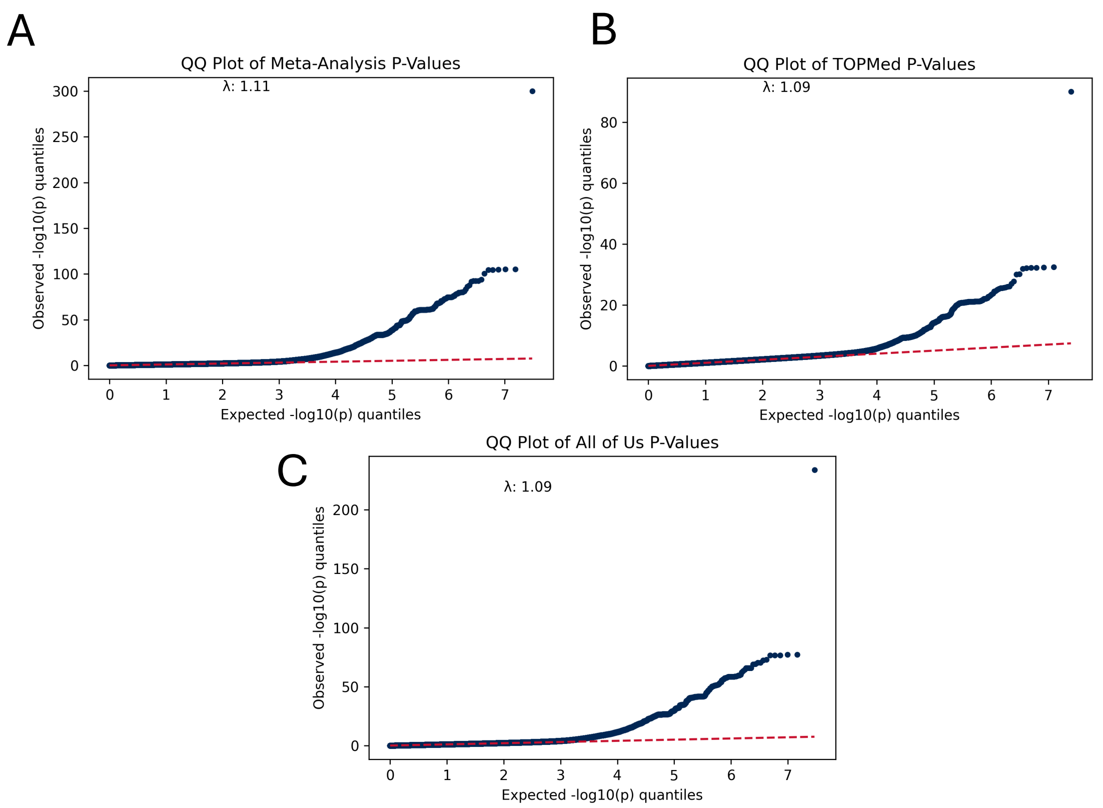


**Supplementary Figure 3: Quantile-Quantile Plots for the Genome Wide Associations Studies.** a) The meta-analysis Q-Q plot with the genomic inflation lambda (N=181,568). b) The TOPMed Q-Q plot with the genomic inflation lambda (N=86,017). c) The All of Us Q-Q plot with the genomic inflation lambda (N=95,551).

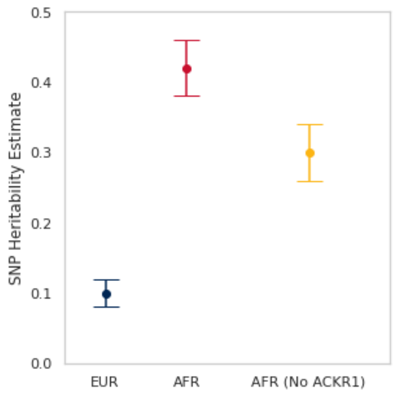


**Supplementary Figure 4: Differential in heritability without ACKR1 in AA.** SNP Heritability estimates for EUR ($h^2_{SNP}$ ~ 0.1, SD ~ 0.02), AFR (total $h^2_{SNP}$ ~ 0.42, SD ~ 0.04), without the ACKR1 locus ($h^2_{SNP}$ ~ 0.30, SD ~ 0.039) were calculated by the SumHer BLD-LDAK model using GWAS summary statistics (EUR N = 57,392, AFR N = 33,636).

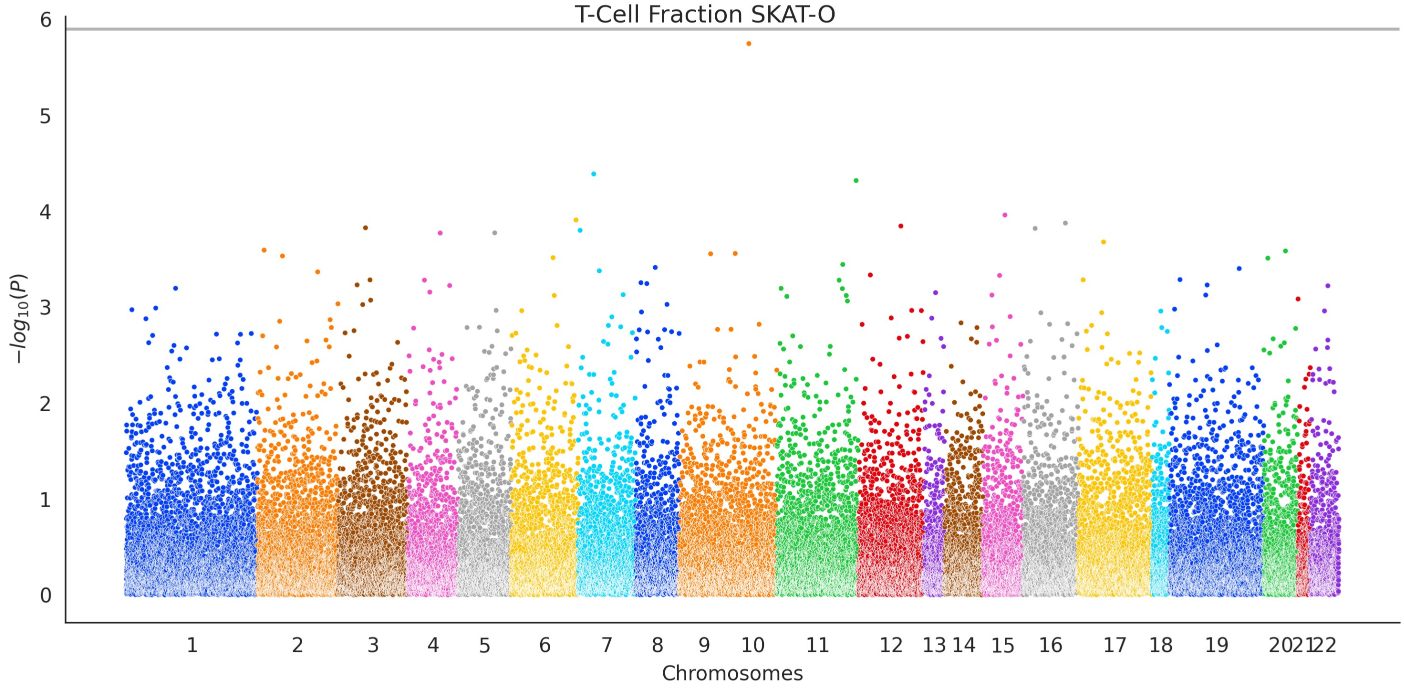


**Supplementary Figure 5: Rare-variant burden analysis using REGENIE pipeline and annotation masks.** The REGENIE pipeline was employed to perform a rare-variant omnibus burden test utilizing five annotation masks. This is a two-sided statistical test. None of the variant masks exhibited a significant association with T-cell fraction. However, a stop-gain mask within the TBATA gene approached a significant association with reduced T-cell fraction ($p = 2x10^{-6}$) (N = 85,988).

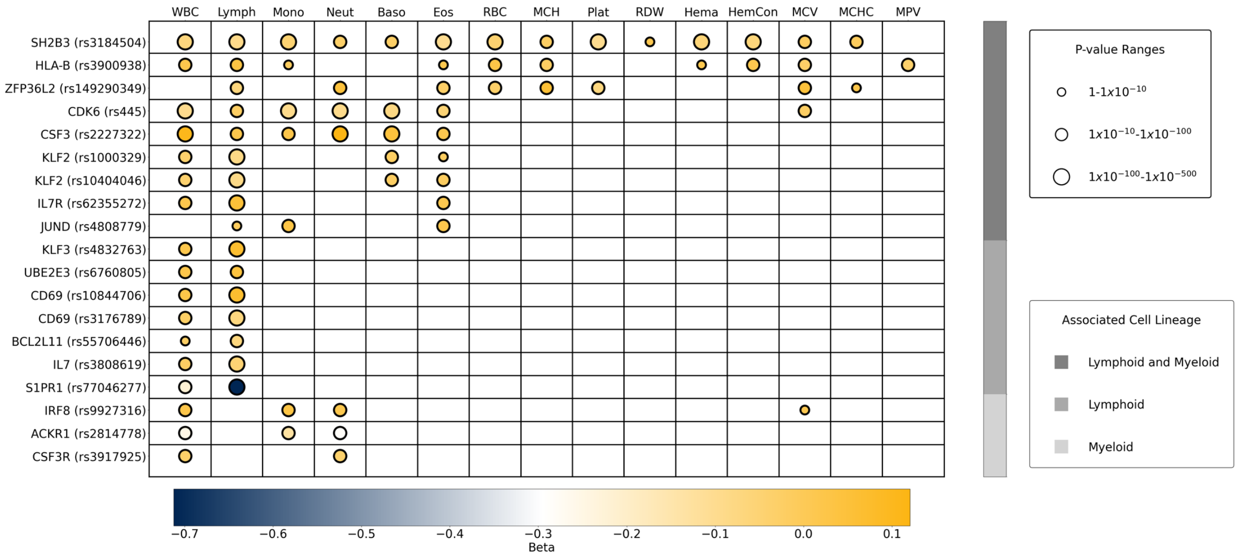


**Supplementary Figure 6: Eight blood cell counts, and seven blood indices were significantly associated with prioritized or lead variants in the Chen et al., 2020 paper.** Prioritized variants from the TOPMed single-variant association study and lead variants from the multi-cohort meta-analysis were queried from the Open Targets single-variants PheWAS. All measurement associations were previously reported as genome-wide significant ($5x10^{-8}$) in Chen et al., 2020. The variants were sorted and classified based on their association with cells from one or more of the hematopoietic lineages.

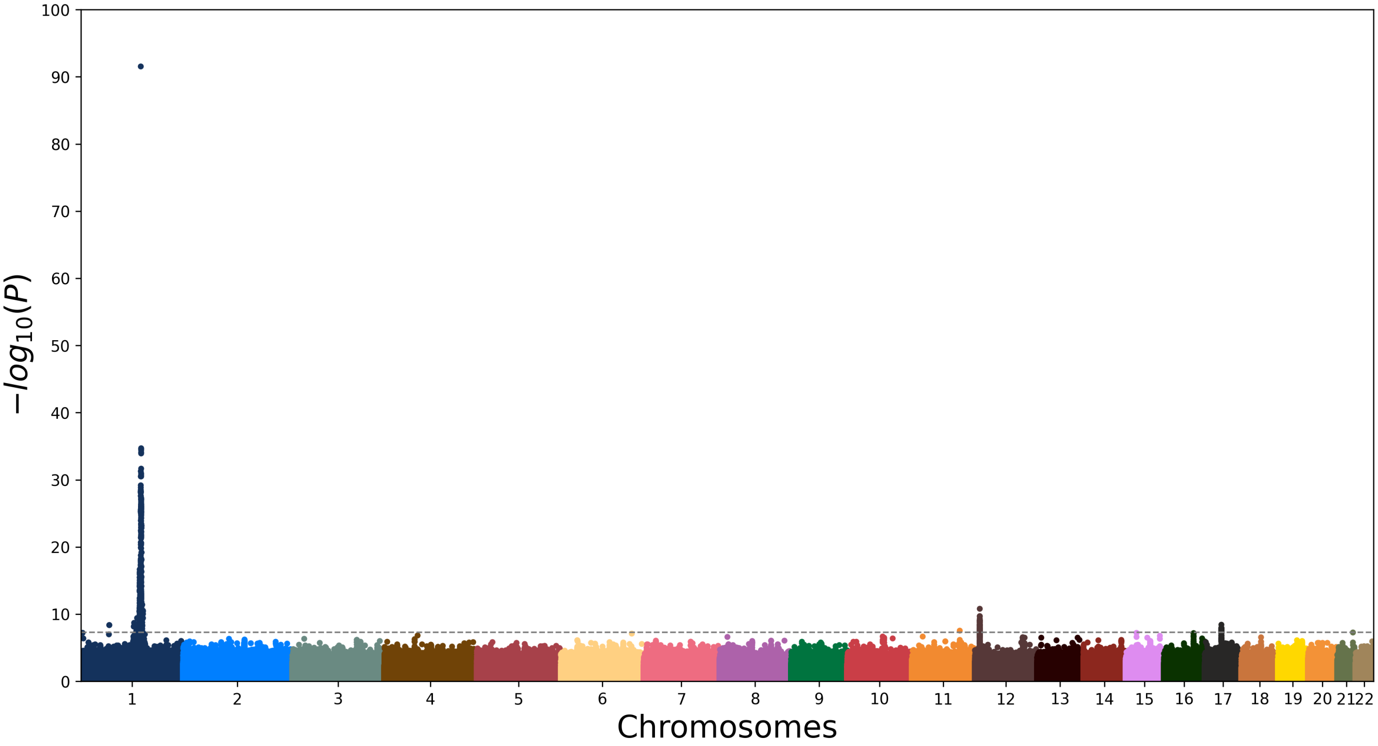


**Supplementary Figure 7: Mean lymphocyte/neutrophil count ratio Genome-wide Association Study (GWAS) using the All of Us.** Mean lymphocyte and neutrophil counts were generated. Using the ratio of these metrics, a two-side association test was performed using REGENIE v3.2 (N=20,847). The dashed grey line represents 5 x $10^{-8}$ our Bonferroni multiple-hypothesis correction p-value threshold for significance.

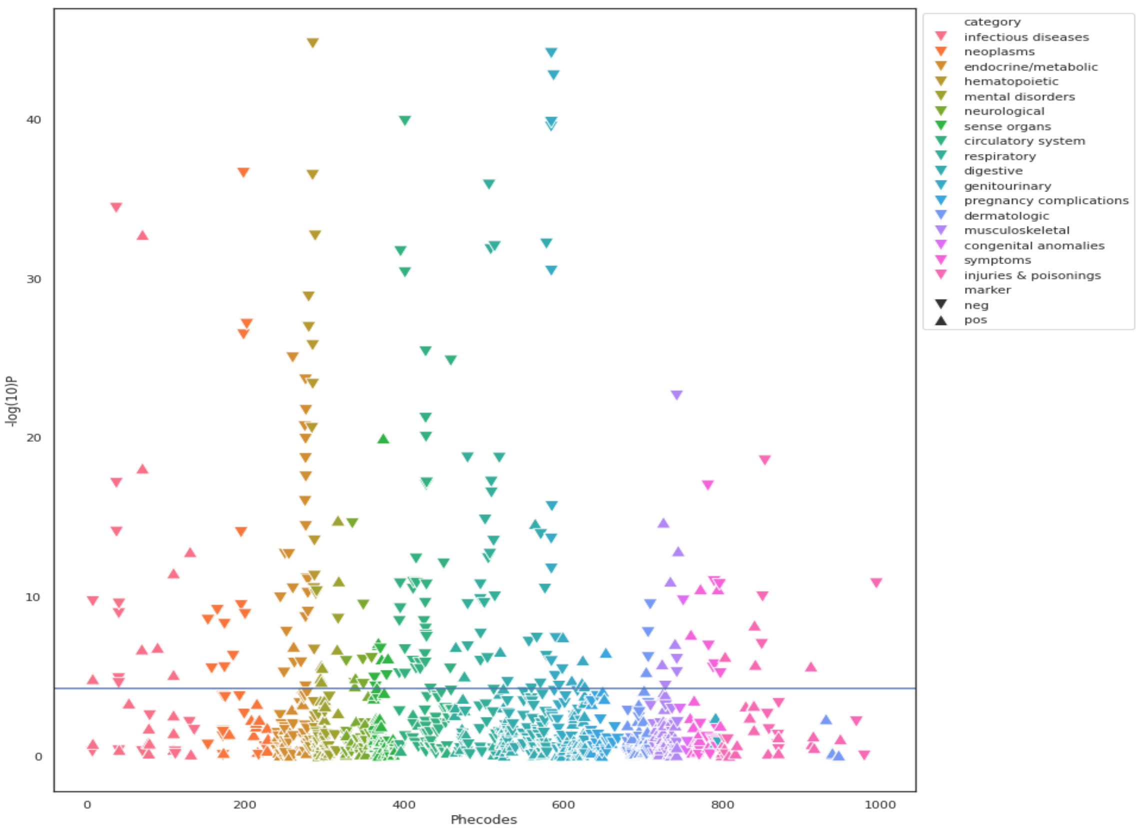


**Supplementary Figure 8: T-Cell Fraction Phenome-wide Association Study (PheWAS) using the All of Us dataset.** ICD9 and ICD10 codes from the All of Us Electronic Health Records (EHR) were converted to phecodes for a cohort of 69,409 individuals. Logistic regression analysis, a two-sided test, was employed to assess the relationship between T-cell fraction and phecodes. More than 100 phecodes exhibited statistically significant associations with T-cell fraction. The phecode classification categories are distinguished by different colors, while the direction of the arrows denotes the beta direction, either positive (∆) or negative (∇). The blue line represents our Bonferroni multiple-hypothesis correction p–value threshold for significance.

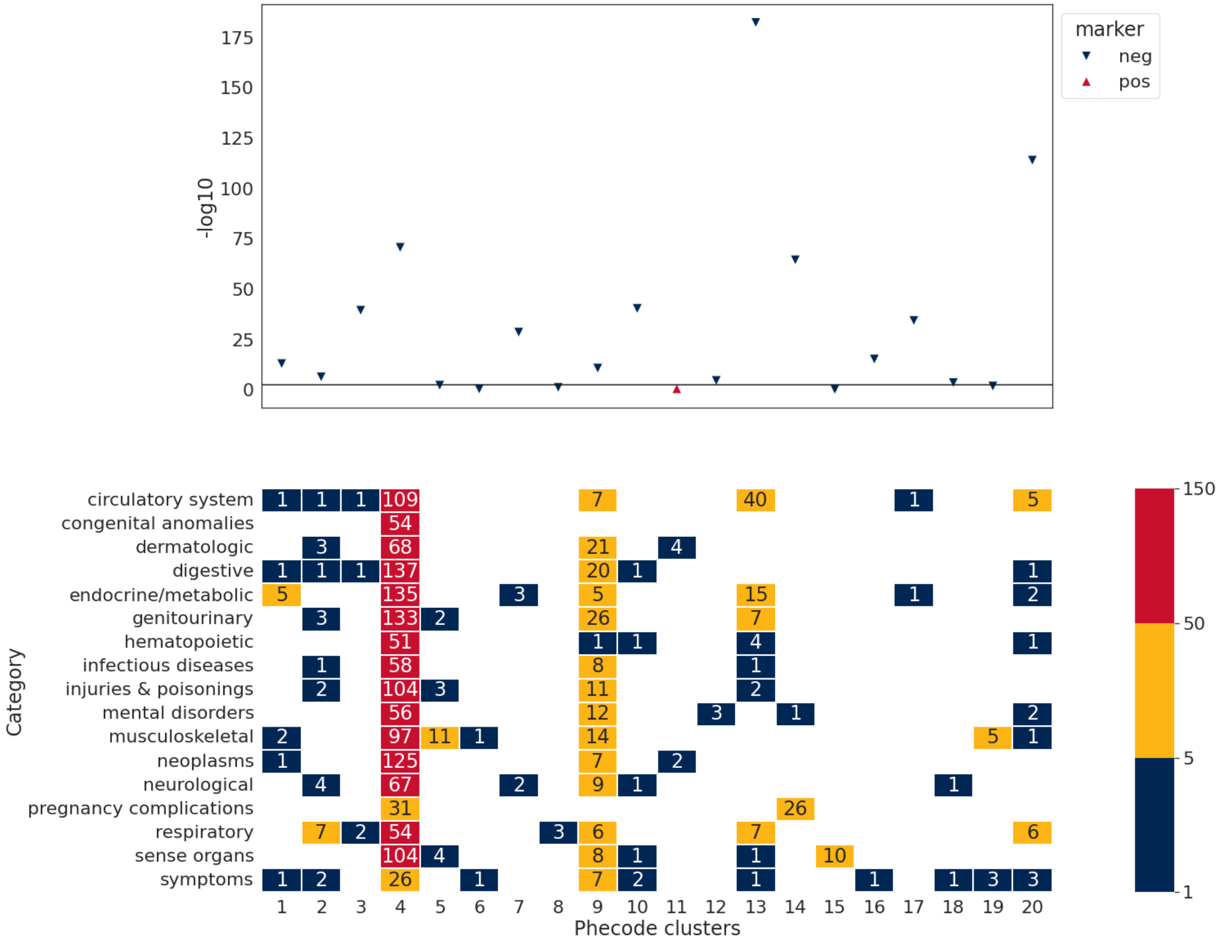


**Supplementary Figure 9: Clustered Phecodes Analysis of T-Cell Fraction using All of Us dataset.** All phecodes derived from the ICD9 and ICD10 billing codes for the 69,409 individuals in the Electronic Health Records (EHR) were subjected to feature agglomeration clustering. Associations between the clusters and T-cell fraction were evaluated using ordinary least squares regression, a two-sided test. Fifteen phecode clusters displayed significant associations with T-cell fraction. The beta direction is indicated, either positive (∆) or negative (∇), as well as by color for each cluster. The black line in the top panel represents our Bonferroni multiple-hypothesis correction p–value threshold for significance.

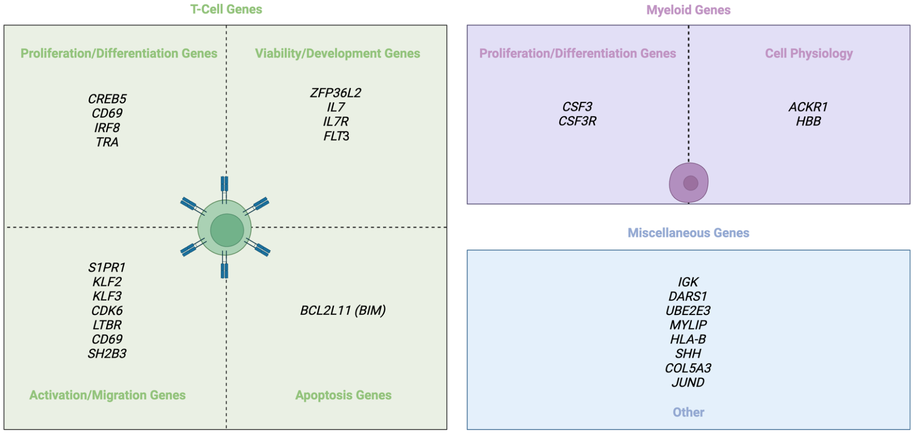


**Supplementary Figure 10: Division of the function of genes identified through the T-cell fraction meta-analysis.** Genes in the green box have known functions in T-cell biology. Genes in the purple box have known functions related to the myeloid lineage. Genes in the blue box either have known functions related to other blood/immune cells or unrelated functions. Created with BioRender.com released under a Creative Commons Attribution-NonCommercial-NoDerivs 4.0 International license
