# Supplementary material for: Genetic determinants and phenotypic consequences of blood T-cell proportions in 207,000 diverse individuals
Source: Nat Commun. 2024 Aug 7;15:6732. doi: 10.1038/s41467-024-51095-1 (PMC11306580; doi:10.1038/s41467-024-51095-1)
Supplement: Supplementary file 3 — Description of Additional Supplementary Files [file 41467_2024_51095_MOESM3_ESM.pdf]

Description of Additional Supplementary Files

File Name: Supplementary Data 1
Description: **Pearson correlations between WGS TCRA T-cell Fraction and 14 harmonized blood traits.** Pearson correlations between WGS TCRA T-cell Fraction and 14 harmonized blood traits demonstrate expected positive correlation with lymphocyte counts and the lymphocyte to neutrophil counts ratio. Negative correlations with other phenotypes further support evidence that our phenotype is a proportion of blood cells.

File Name: Supplementary Data 2
Description: **Pearson correlations between WGS TCRA T-cell Fraction and 11 blood traits available through the All of Us EHR.** All values included were measured on the same day as the blood draw used to generate the sequencing data. Strong positive correlations with lymphocyte count and the ratio of lymphocyte count to neutrophil count, suggest that our estimate does represent a proportion of the cells. The strong negative correlation with neutrophil count and white blood cell count, provide additional evidence that our metric is a proportion of blood cells present in participants.

File Name: Supplementary Data 3
Description: **90 genes used to validate T-Cell ExTRECT in WGS.** 90 random genes were selected to confirm that coverage at the *TRA* region was consistent with sequencing coverage throughout the genome.

File Name: Supplementary Data 4
Description: **T-cell fractions estimated in TOPMed associated with 5 principal components.** T-cell fraction was significantly associated with ancestry principal components one, four, seven, eight, and nine. Principal components one, four, and seven were useful for stratifying individuals with African Ancestry, South Asian ancestry, and the gradient of individuals with European ancestry (N=86,017). An ordinary least squares regression, a two-sided test, with Bonferroni multiple-hypothesis correction was applied.

File Name: Supplementary Data 5
Description: **14 putatively causal variants across 11 loci from the TOPMed single variant association analysis.** Variants were identified using a two-side association test performed using SAIGE. Significance was assigned to variants that exceeded our 5×10^{-8}, our Bonferroni multiple-hypothesis correction p-value threshold

File Name: Supplementary Data 6
Description: **Significant laboratory values identified through the LabWAS.** The test performed was two-sided and a Bonferroni multiple-hypothesis correction was applied.

File Name: Supplementary Data 7
Description: **Regression associations with phecode clusters.** Ordinary least squares regression, a two-sided test, was used to evaluate the association between the phecode clusters and T-cell fraction. A Bonferroni multiple-hypothesis correction was applied. Adjustments were made for age, sex, and the first ten ancestry principal components. Cluster 4 is primarily composed of phecodes related to infections, cluster 13 is primarily composed of hematopoietic measures and codes related to the endocrine system, cluster 14 is entirely pregnancy-related phecodes, and cluster 20 is composed of phecodes primarily in the circulatory and respiratory system categories (N =69,409).

File Name: Supplementary Data 8
Description: **Cohorts included in the TOPMed T-cell fraction estimation**
