# Supplementary material for: Genetic determinants and phenotypic consequences of blood T-cell proportions in 207,000 diverse individuals
Source: Nat Commun. 2024 Aug 7;15:6732. doi: 10.1038/s41467-024-51095-1 (PMC11306580; doi:10.1038/s41467-024-51095-1)
Supplement: Supplementary file 5 — Reporting Summary [file 41467_2024_51095_MOESM5_ESM.pdf]

nature portfolio

Corresponding author(s): Bick, A. G.

Last updated by author(s): Jul 1, 2024

Reporting Summary

Nature Portfolio wishes to improve the reproducibility of the work that we publish. This form provides structure for consistency and transparency in reporting. For further information on Nature Portfolio policies, see our Editorial Policies and the Editorial Policy Checklist.

Statistics

For all statistical analyses, confirm that the following items are present in the figure legend, table legend, main text, or Methods section.

n/a	Confirmed	
☐	☒	The exact sample size (*n*) for each experimental group/condition, given as a discrete number and unit of measurement
☒	☐	A statement on whether measurements were taken from distinct samples or whether the same sample was measured repeatedly
☐	☒	The statistical test(s) used AND whether they are one- or two-sided<br>*Only common tests should be described solely by name; describe more complex techniques in the Methods section.*
☐	☒	A description of all covariates tested
☐	☒	A description of any assumptions or corrections, such as tests of normality and adjustment for multiple comparisons
☐	☒	A full description of the statistical parameters including central tendency (e.g. means) or other basic estimates (e.g. regression coefficient) AND variation (e.g. standard deviation) or associated estimates of uncertainty (e.g. confidence intervals)
☐	☒	For null hypothesis testing, the test statistic (e.g. *F*, *t*, *r*) with confidence intervals, effect sizes, degrees of freedom and *P* value noted<br>*Give P values as exact values whenever suitable.*
☐	☒	For Bayesian analysis, information on the choice of priors and Markov chain Monte Carlo settings
☒	☐	For hierarchical and complex designs, identification of the appropriate level for tests and full reporting of outcomes
☐	☒	Estimates of effect sizes (e.g. Cohen's *d*, Pearson's *r*), indicating how they were calculated

Our web collection on statistics for biologists contains articles on many of the points above.

Software and code

Policy information about availability of computer code

Data collection	N/A
Data analysis	Software for generating T-cell fractions include samtools (v1.14) for estimating allele depth, and T-cellExTRECT (v1.0.1) for generating the fractions. Software for data analysis includes<br>TOPMed: Python 3.7.12, NumPy (v1.21.2), sklearn (v0.24.2), and statsmodels (v0.13.0). Plotting and analysis in Python made used of the Seaborn (v0.11.2), Scipy (v1.7.1), Matplotlib (v3.4.3), and Pandas (v1.3.3) packages.<br>All of Us: Python 3.7.16. sklearn (v1.0.2) and statsmodels (v0.13.5). Plotting and analysis in Python made use of the Seaborn (v0.12.1), Scipy (v1.7.3), Matplotlib (v3.5.1), NumPy (v1.21.6), and Pandas (v1.3.5) packages. PsymPy (v0.3.13) was used to perform the age and sex match for the pregnancy analysis.<br>Plink v1.9 -variant filtering for GWAS and meta-analysis<br>REGENIE 3.2-All of Us GWAS and TOPMed SKAT-O<br>SAIGE-TOPMed GWAS<br>PolygenicRiskScores.jl v1 for generating the EUR only polygenic risk scores<br>SumHER BLD-LDAK model v5.2

For manuscripts utilizing custom algorithms or software that are central to the research but not yet described in published literature, software must be made available to editors and reviewers. We strongly encourage code deposition in a community repository (e.g. GitHub). See the Nature Portfolio guidelines for submitting code & software for further information.

Data

Policy information about availability of data

All manuscripts must include a data availability statement. This statement should provide the following information, where applicable:

- Accession codes, unique identifiers, or web links for publicly available datasets
- A description of any restrictions on data availability
- For clinical datasets or third party data, please ensure that the statement adheres to our policy

The GWAS summary statistics generated in this study have been deposited in this Zenodo repository (https://doi.org/10.5281/zenodo.12582912). All TOPMed WGS used in this analysis are available to researchers through the NHLBI BioData Catalyst ecosystem. Please see supplementary table 10 for relevant accession numbers. Access to individual-level data from the All of Us research program is available to researchers whose institution has signed a data use agreement with All of Us. All of Us provides a publicly available data browser (https://databrowser.researchallofus.org/) containing aggregate-level participant data for users to explore the available data, including genomic variants. Electronic health records (EHR) data, used for phenotyping, belongs to the registered tier dataset. Whole-genome sequencing data belongs to the controlled tier dataset, which requires additional training to access. The All of Us estimated T-cell fractions are available in a public workspace and are available upon completion of the relevant training necessary to obtain individual level data. The TOPMed T-cell fractions used in this study are available in Github available here (https://github.com/bicklab/tcf_paper).

Research involving human participants, their data, or biological material

Policy information about studies with human participants or human data. See also policy information about sex, gender (identity/presentation), and sexual orientation and race, ethnicity and racism.

Reporting on sex and gender	All analyses used self-reported sex at birth.
Reporting on race, ethnicity, or other socially relevant groupings	All analyses used genetically estimated ancestry. Ancestry principal components were included in all predictive analyses.
Population characteristics	The NHLBI TOPMed program consists of 51 studies. 109,019 participants have whole-genomes available for analysis. ~70% of the studies are focused on lung and heart phenotypes. ~60% of the participants have primarily European ancestry and 40% have non-European ancestry.<br><br>The All of Us Research program is a biobank. 98,590 participants have undergone whole-genome sequencing. ~60% of participants have primarily European ancestry and ~40% have non-European ancestry.<br><br>Polygenic scores were generated for 72,828 participants with primarily European ancestry in the BioVU Biobank.<br><br>Age and self-reported sex at birth were used for all predictive analyses. Median BMI was also used in the LabWAS.
Recruitment	N/A: We were not involved in any recruitment efforts for either cohort.
Ethics oversight	Informed consent for All of Us participants was obtained either in person or through an eConsent platform approved of by the All of Us IRB. The individual studies of TOPMed each have their own approval, related to the IRB's at their respective institutions, and are supported by the NHLBI. Each of the studies obtained informed consent from each participant.

Note that full information on the approval of the study protocol must also be provided in the manuscript.

Field-specific reporting

Please select the one below that is the best fit for your research. If you are not sure, read the appropriate sections before making your selection.

[x] Life sciences [ ] Behavioural & social sciences [ ] Ecological, evolutionary & environmental sciences

For a reference copy of the document with all sections, see nature.com/documents/nr-reporting-summary-flat.pdf

Life sciences study design

All studies must disclose on these points even when the disclosure is negative.

Sample size	TOPMEd: 109,019 with WGS data and 86,017 with age and sex data.<br>All of Us: 98,590 with WGS data and 95,551 with age and sex data. 69,409 individuals with EHR data.<br>BioVU: 72,828 with genetic and EHR data<br>The number of datasets involved in this study was determined based on the available whole-genomes at the time of analysis. The main cohorts were also selected because they were similar in make-up and include individuals with diverse ancestries.
Data exclusions	TOPMed and All of US: For GWAS participants must have age, sex, and genetic ancestry principal components.
Replication	All of Us served as our replication cohort. The findings that there are variants associated with T-cell fraction indicated to these authors that

	this was a successful replication.
Randomization	TOPMed, All of Us, BioVU: N/A (observational study). Relevant variables were controlled for in all statistical models.
Blinding	TOPMed, All of Us, BioVU: N/A (observational study)

Reporting for specific materials, systems and methods

We require information from authors about some types of materials, experimental systems and methods used in many studies. Here, indicate whether each material, system or method listed is relevant to your study. If you are not sure if a list item applies to your research, read the appropriate section before selecting a response.

Materials & experimental systems

n/a	Involved in the study
☒	☐ Antibodies
☒	☐ Eukaryotic cell lines
☒	☐ Palaeontology and archaeology
☒	☐ Animals and other organisms
☒	☐ Clinical data
☒	☐ Dual use research of concern
☒	☐ Plants

Methods

n/a	Involved in the study
☒	☐ ChIP-seq
☒	☐ Flow cytometry
☒	☐ MRI-based neuroimaging

Plants

Seed stocks	*Report on the source of all seed stocks or other plant material used. If applicable, state the seed stock centre and catalogue number. If plant specimens were collected from the field, describe the collection location, date and sampling procedures.*
Novel plant genotypes	*Describe the methods by which all novel plant genotypes were produced. This includes those generated by transgenic approaches, gene editing, chemical/radiation-based mutagenesis and hybridization. For transgenic lines, describe the transformation method, the number of independent lines analyzed and the generation upon which experiments were performed. For gene-edited lines, describe the editor used, the endogenous sequence targeted for editing, the targeting guide RNA sequence (if applicable) and how the editor was applied.*
Authentication	*Describe any authentication procedures for each seed stock used or novel genotype generated. Describe any experiments used to assess the effect of a mutation and, where applicable, how potential secondary effects (e.g. second site T-DNA insertions, mosiacism, off-target gene editing) were examined.*
